# Transposons Acting as Competitive Endogenous RNAs: In-Silico Evidence from Datasets Characterised by L1 Overexpression

**DOI:** 10.3390/biomedicines10123279

**Published:** 2022-12-17

**Authors:** Mauro Esposito, Nicolò Gualandi, Giovanni Spirito, Federico Ansaloni, Stefano Gustincich, Remo Sanges

**Affiliations:** 1 Computational Genomics Laboratory, Area of Neuroscience, Scuola Internazionale Superiore di Studi Avanzati (SISSA), 34136 Trieste, Italy; 2 CMP3vda, via Lavoratori Vittime del Col Du Mont 28, 11100 Aosta, Italy; 3 Central RNA Laboratory, Istituto Italiano di Tecnologia, 16132 Genova, Italy

**Keywords:** transposons, LINE1, miRNA, ceRNA, p53

## Abstract

LINE L1 are transposable elements that can replicate within the genome by passing through RNA intermediates. The vast majority of these element copies in the human genome are inactive and just between 100 and 150 copies are still able to mobilize. During evolution, they could have been positively selected for beneficial cellular functions. Nonetheless, L1 deregulation can be detrimental to the cell, causing diseases such as cancer. The activity of miRNAs represents a fundamental mechanism for controlling transcript levels in somatic cells. These are a class of small non-coding RNAs that cause degradation or translational inhibition of their target transcripts. Beyond this, competitive endogenous RNAs (ceRNAs), mostly made by circular and non-coding RNAs, have been seen to compete for the binding of the same set of miRNAs targeting protein coding genes. In this study, we have investigated whether autonomously transcribed L1s may act as ceRNAs by analyzing public dataset in-silico. We observed that genes sharing miRNA target sites with L1 have a tendency to be upregulated when L1 are overexpressed, suggesting the possibility that L1 might act as ceRNAs. This finding will help in the interpretation of transcriptomic responses in contexts characterized by the specific activation of transposons.

## 1. Introduction

Transposable elements (TE) are interspersed and repeated DNA elements that can change their position within the genome. Based on the mechanism used for the transposition, they can be divided into DNA transposons and retrotransposons. The first ones are excised from the original genomic locus and inserted into a new position with the activity of a transposase enzyme. Retrotransposons instead can generate new copies by reverse transcribing their RNA molecules [1]. Among the several TE families, LINE L1 is the only one that contains autonomous elements considered to be still active in the human genome. L1 retrotransposons account for 17% of the human genome [2]. Despite the human genome being composed of more than 500,000 L1 fragments, the vast majority of these are inactive since they underwent 5′ truncations and accumulation of mutations [3]. A full-length L1 retrotransposon is 6 kb long and it is composed of a 5′UTR with an internal promoter, two open reading frames (ORF1 and ORF2) that provide the retrotransposition proteins, and a 3′UTR region. The human genome contains more than 5000 full-length elements and 100 out of 150 copies that are still potentially capable to mobilize [4] since they have intact and functional ORFs. It has been observed that L1 novel insertions are not randomly distributed in the genome [5]. This has suggested an underlying evolutionary force that guided the co-option of these elements for beneficial cellular functions [6]. The positive selection of L1 elements in specific genomic regions might be linked to their capability to influence the expression of nearby genes. L1 insertions close to a given gene can provide promoters and other cis-regulatory elements [7] resulting in changes of transcriptional levels in the flanking *loci*. Conversely, insertions that induce alternative splicing events [8] or introduce miRNA target sites in 3′UTR regions [9] are examples of L1 contribution to post-transcriptional regulation. Despite selected beneficial functions, deregulated L1 activity can be detrimental to the cell and is involved in diseases, e.g., cancer and neurodegenerative diseases [10]. As a result, L1s activity is repressed at all stages of the retrotransposition process through several cellular defense mechanisms. At the genomic level, L1 loci are generally found to be inaccessible because DNA methylation and repressive epigenetic marks decrease the accessibility to the transcriptional machinery [11,12]. In addition, especially in germ cells, the Piwi-interacting RNA (piRNA) pathway acts at post-transcriptional levels to degrade L1s RNA or to inhibit its translation. The interfering activity is mediated by a complex of the small non-coding RNA piRNA and the Argonaute (Ago) proteins of the PIWI subfamily [13]. In the final stages of retrotransposition, new L1 integration sites are continuously targeted by DNA repair mechanisms for preventing the insertion of new elements [14]. Despite all these mechanisms, we do not yet have a comprehensive understanding of all the regulatory layers affecting L1 expression and how their deregulation can impact cells’ homeostasis.

One of the main post-transcriptional regulatory mechanisms acting in somatic cells is represented by miRNAs, a class of small non-coding RNAs that have a fundamental role in gene expression control [15]. In the canonical pathway, miRNA genes are transcribed into primary miRNA (pri-miRNA) and processed into precursor miRNA (pre-miRNA) through the Microprocessor complex [16]. The pre-miRNA is then exported to the cytoplasm by Exportin 5 and finally processed in the mature miRNA duplex by the endonuclease Dicer [17]. The loading of the miRNA guide strand into the Argonaute protein allows the formation of the miRNA-induced silencing complex (RISC) [18]. This complex allows the binding of the specific target transcript with the loaded miRNA and then proceeds to the following steps of the process. In detail, the fate of target transcripts depends mostly on the binding between specific sequences located in the 3′ UTR regions and the miRNA. Although still not completely understood, it is believed that the degree of miRNA-target complementarity might determine whether the target transcript undergoes translational inhibition or degradation [19]. The activity of the Agonaute protein is essential for the choice of the fate. Indeed, a full pairing between the target site and the 5′ seed region (nucleotides 2–8) of the miRNA activates the endonuclease function of Argonaute, allowing mRNA slicing [20]. On the other hand, a limited miRNA-target base pairing triggers a slicer-independent pathway, leading to the recruitment of factors involved in translational inhibition or deadenylation followed by transcript decay [21,22].

In the last few years, knowledge became available on the existence of miRNA-based mechanisms used by the cell to inhibit the translation of L1 elements. In 2015, Hamdorf et al. hypothesized that miRNA can protect non-germ cells from L1 retrotransposition [23]. Particularly, they demonstrated that miR-128 can bind the ORF2 region of L1 RNAs, inducing the degradation of the transcripts. In a more recent publication, Tristàn-Ramos et al. [24] demonstrated that let-7 miRNAs bind to the ORF2 region of L1 RNAs, inhibiting their translation and altering the protein amount needed for the retrotransposition to happen.

RNA molecules that regulate transcript levels of other genes through the competition for the binding of the same pool of miRNAs have also been discovered [25]. This system formed by competitive endogenous RNAs (ceRNAs) creates crosstalk between different coding and non-coding transcripts. The first evidence of this mechanism was observed in the Poliseno study [26] which demonstrated that overexpressed 3′UTR of PTENP1 pseudogene was able to act as ceRNA for miRNAs. Indeed, the sequestering of miRNAs by PTENP1 pseudogene transcript resulted in the increase of the levels of the functional tumor suppressor PTEN transcript. The involvement of an apparent non-functional gene in this mechanism drew attention to long non-coding RNAs (lncRNAs). These are ideal candidate molecules for a widespread ceRNA mechanism given their activity as regulatory RNAs since they lack protein encoding open reading frames [25]. In this context, the RIDL hypothesis [27] proposed that the enrichment of TEs embedded in lncRNA transcripts provides functional domains, allowing interactions with proteins and/or other nucleic acids. Accordingly, the functional properties of lncRNAs may strictly depend on their different domain combinations. As a representative example, the antisense lncRNAs *AS Uchl1* showed the ability to increase translation of its sense target gene through the activity of an effector domain made by an embedded TE and an antisense sequence as binding domain [28]. The antisense BACE1-AS is a clear example of a lncRNA in which embedded TEs provide miRNA target sites to sequester and therefore subtract miRNAs from their canonical targets [29].

Here, we aim at performing a first evaluation of the possibility that TEs might generally sequester miRNAs impacting on regulatory mechanisms in human cells. To this end, we carried out transcriptomic analyses of different cellular conditions known to undergo L1s overexpression [30,31,32,33]. We observed that genes sharing a high number of miRNA target sites with overexpressed L1s are more upregulated with respect to genes sharing a lower number of miRNA target sites with L1s. Our results are consistent with a possible ceRNAs activity mediated by L1 retrotransposon transcripts. These findings might help in the interpretation of transcriptional responses to the deregulation of TEs expression.

## 2. Materials and Methods

### 2.1. Data Collection and Pre-Processing

To explore how regulatory mechanisms control L1 transcript levels in human cells, we took advantage of different publicly available datasets overexpressing L1s. The dataset from Jonsson et al. [30] is composed of poly-A RNA-seq data generated from embryo-derived human neural epithelial-like stem cell line Sai2. In this study, the authors produced 3 controls and 3 *DNMT1* samples in which *LacZ* and *DNMT1* genes were respectively knocked out with CRISPR-Cas9 technology. We retrieved paired-end raw FASTQ files from the ENA-EBI database (PRJNA420729 accession code). For validating the reliability of our method to analyze non-autonomous L1 transcription, we used the Marasca et al. [31] dataset. It is composed of total-RNA derived from quiescent naive CD4^+^ T cells and naive CD4^+^ T cells activated with anti-CD3-antiCD28 beads (3 individuals, 24 total sequencing). The download of paired-end raw FASTQ files was made from the ENA-EBI database (PRJEB41930 accession code). For testing the miRNA-L1 expression levels association, we used RNA-seq and short RNA-seq data produced by the Geuvadis Project [34]. Quantification of miRNAs was retrieved from the ArrayExpress [35] repository while raw reads were retrieved for the TE quantification from ENA-EBI database (PRJEB3366 accession code). Aiming to investigate a cellular context into which L1 was artificially overexpressed, we took advantage of the publicly available RNA-seq data derived from the Ardeljan et al. study [32]. In this work, the authors performed RNA sequencing of human *Retinal Pigment Epithelial Cells* (RPE) encoding a doxycycline-inducible (Tet-On) codon-optimized L1 (ORFeus) [36] or luciferase as control. The two groups of cultures were sequenced in triplicates and the paired-end raw files were made publicly available in the ENA-EBI database (PRJNA491205 accession code). For exploring other cellular contexts in which L1s should be deregulated, we analyzed the Deneault et al. [33] polyA RNA-seq data in which, the *ATRX* gene was knocked-out in reprogrammed human induced pluripotent stem cells (iPSC) and iPSC differentiated in neuronal cells. The entire dataset composed of 12 controls and 8 treated samples was retrieved from the ENA-EBI database (PRJNA422099 accession code). The quality assessment of all the retrieved reads was performed with FastQC [37].

### 2.2. Analysis of Locus-Specific TE Expression

To analyze the TE transcriptome, we used the *SQuIRE* [38] software for quantifying the locus-specific TE expression starting from RNA-seq reads. The customized parameter *--build hg19* was used to allow the download of the needed files referring to the human reference genome *hg19*. Once we obtained the quantifications, we performed the differential expression analysis of TE using the *R* package *DESeq2* [39], classifying as differentially expressed TE the elements showing p-adjusted < 0.05 and |fold change| > 1.5.

### 2.3. Detection of L1 Autonomous Transcription

To understand if an L1 up-regulation is caused by the autonomous transcription of L1 loci, we developed a specific *R* pipeline that analyzes paired-end reads of an RNA-seq experiment. In our method, reads are aligned on the L1 consensus sequence [40] with *BWA* [41] (*mem* command in default parameters). Then, not primary and supplementary alignments are discarded using *Samtools* [42] (*-F 2304*). The remaining fragments (referred to as a pair of reads) are filtered out if at least one read of the pair has more than 20% of nucleotides that are not perfectly matched on the L1 consensus sequence. At this point, we label the fragments that are completely aligned inside the L1 elements as “*Inside*” fragments and the fragments with exclusively one read aligned inside the L1 as “*Outside*” fragments. These sets of fragments are then aligned on the human reference genome *hg19* with *BWA* [41] (*mem* command in default parameters). On the resulting BAM file, we apply the same previously described filters. In the final step, we use *Bedtools* [43] (*intersect* command) to intersect the mapping coordinates of the reads with a BED file containing the L1 elements annotated on the human reference genome (*RepeatMasker* [44] track retrieved from UCSC Table Browser [45]). Fragments with at least one read aligned on an annotated L1 element are kept into account for the final calculation. For each sample, the ratio between the number of *Inside* and *Outside* fragments is used as an indicator for the L1 autonomous transcription level in all the group comparisons except for the *ORFeus* model in which the same ratio was calculated before the genome mapping step.

### 2.4. Gene Expression Analysis

To investigate the gene expression levels, we aligned the FASTQ RNA-seq reads to the human reference genome *hg19* (FASTA file of primary assembly retrieved from *Ensembl* [46]) using *STAR* [47]. The indexing step was performed giving --*sjdbGTFfile* argument the GRCh37.87 GTF file retrieved from *Ensembl*. The –sjdbOverhang argument was instead set to *max(ReadLength)-1* for the different datasets. Then, the mapping was performed with the parameters *--quantMode GeneCounts* and *--twopassMode Basic*. After the generation of BAM files, the counting of mapped reads for each gene was obtained with *HTSeq* [48] (*htseq-count* command with the arguments *-t exon -i gene_id).* Finally, the differential gene expression analysis was performed using *R* package *DESeq2* [39] classifying, as differentially expressed, the genes showing p-adjusted < 0.05 and |fold change| > 1.5.

### 2.5. Functional Enrichment Analysis

To explore the transcriptomic changes in the *DNMT1* model, the differentially expressed genes were divided into upregulated and downregulated according to previous thresholds. The two separated groups were then used as input to perform the enrichment analysis with the *R* package *gProfiler2* [49] (*exclude_iea = T, user_threshold = 0.1* and *correction_method = “fdr”*). The background (*custom_bg* argument) used for the analysis was composed of genes with at least 5 mapped reads in at least 50% of the samples. Gene sets composed of more than 1000 genes were filtered out since they described too many general cellular processes. For the final exploration, gene sets with at least 10 enriched query genes and FDR ≤ 0.1 were considered significant.

### 2.6. Overlap Analysis

To study how L1 overexpression can impact regulatory gene networks, we performed an overlap analysis between genomic coordinates of L1s and protein-coding genes. For this analysis, we used the GRCh37.87 GTF annotation file retrieved from *Ensembl* [46]. From this, we selected all the exonic and UTR annotations of protein-coding genes. Then, we used *Bedtools merge* [43] for collapsing together the features belonging to different transcripts of the same gene. *Bedtools subtract* was used to exclude both the UTR annotations from the exons and to generate the intron coordinates for each gene. As a result, we created a BED file containing a single gene model for each protein-coding gene. The models were thus composed of the coordinates of genic structures 5′UTR, exons, introns, and 3′UTR. These coordinates were intersected with L1 coordinates (retrieved from *SQuIRE* [38] output) by using *Bedtools intersect* with “*-s*” argument to force the strandedness. Each genic structure was then classified based on two pieces of information: the up/down-regulation of the belonging gene and the overlap with at least one up/down-regulated L1s. At this point, for each type of genic structure, we calculated the frequency of each possible pair of classifications. For the final calculation of the Z-score, the genic classifications were randomized 1000 times recomputing the calculation of the frequencies each time, which represents the random distribution.

### 2.7. Analysis of miRNA Target Sites Sharing

To determine whether the set of upregulated extragenic L1 elements are sharing miRNA target sites with the 3′UTR of protein-coding genes, we used the BED file of genic structures created in the overlap analysis reported above. From this BED, we selected the 3′UTR regions longer than 30 nucleotides belonging to expressed protein-coding genes. A gene was considered as expressed if it was quantified with at least one mapped read in at least one sample. The upregulated L1 elements were identified with *SQuIRE* [38] as reported above, while the extragenic positioning of these elements was determined by intersecting the genomic coordinates of L1 and features annotated in the GRCh37.87 GTF file. All L1s that did not overlap with any concordant/discordant feature (*Bedtools intersect* with *-s* and *-S* arguments [43]) were considered extragenic. Having selected the 3′UTRs and L1 genomic coordinates, we used *Bedtools getfasta* to retrieve the FASTA file of each element and a custom *Perl* script to identify target sites by looking only for 8-mer seed-matched sites [19] of the entire set of human miRNAs retrieved from *miRBase* database (Release 22.1) [50]. Hence, for each 3′UTR of protein-coding genes, the number of miRNA target sites that were also found in the pool of extragenic upregulated L1s was calculated. T-test was finally used for comparing the number of L1-shared miRNA target sites between up and down-regulated genes.

### 2.8. Identification of miRNAs Sequestered by L1s

To search for miRNAs that are possibly sequestered by L1s, we analyzed miRNAs that were seen to potentially target at least one upregulated extragenic L1. For each of the miRNAs, we identified the targeted protein-coding genes by searching for 8-mer seed-matched sites. The targeted genes were then classified in up or down-regulated based on the previous differential gene expression analysis. At this point, for each miRNA, we performed a proportion test (*prop.test R* function) to compare the proportion of up and down-regulated targeted genes to the proportion of all up and down-regulated genes. miRNAs in which the proportion of targeted upregulated genes was significantly (FDR < 0.1) higher than the proportion of downregulated ones were selected for further analyses.

### 2.9. miRNA-Gene Networks Identification

To explore miRNA-genes interactions, we used the MIENTURNET [51] web-based tool. We provided the miRBase ID list of 117 miRNAs potentially decoyed by L1s. The miRNA-target enrichment analysis was performed with the minimum number of miRNA-target interactions set to 2 and an adjusted p-value (FDR) threshold of 0.1. The interactions categorized by *miRTarBase* [52] as strongly and weakly validated were taken into account. The following enrichment analysis of 57 genes upregulated in the *DNMT1* model was performed with *EnrichR* [53] considering significant the gene set enriched with an FDR < 0.1.

### 2.10. Analysis of miRNA-L1 Expression

To investigate the association between the expression levels of miRNAs and LINE1 elements in Geuvadis dataset [34], we used the quantifications retrieved from ArrayExpress [35] or obtained with SQuIRE [38] respectively. For each sample, the L1 expression level was obtained by summing the DESeq2 [39] normalized counts of elements belonging to the L1HS/L1PA subfamilies and longer than 5000 bp. The sample “NA18861” was discarded since it represented an outlier. The miRNA-L1 expression levels association was analyzed by performing Pearson’s correlation tests.

### 2.11. Analysis of TE Expression at the Consensus Level

In order to investigate the TE transcriptome of the *ORFeus-OE* model, we used the *TEspeX* [54] software. For the analysis, we provided a modified version of TE consensus sequences from the *Dfam* database [55] that includes the L1-ORFeus sequence (https://www.addgene.org/browse/article/28204003/ accessed on 24 August 2021). Furthermore, the annotation files of coding and non-coding transcripts were retrieved from *Ensembl* [46] and referred to the *hg19* version of the human genome. After obtaining the quantifications, we used the *R* package *DESeq2* for differential expression analysis, classifying as differentially expressed TE the subfamilies showing p-adjusted < 0.05 and fold change > |1.5|.

## 3. Results

### 3.1. L1s Are Autonomously Transcribed upon DNMT1-KO

**Figure 1:**
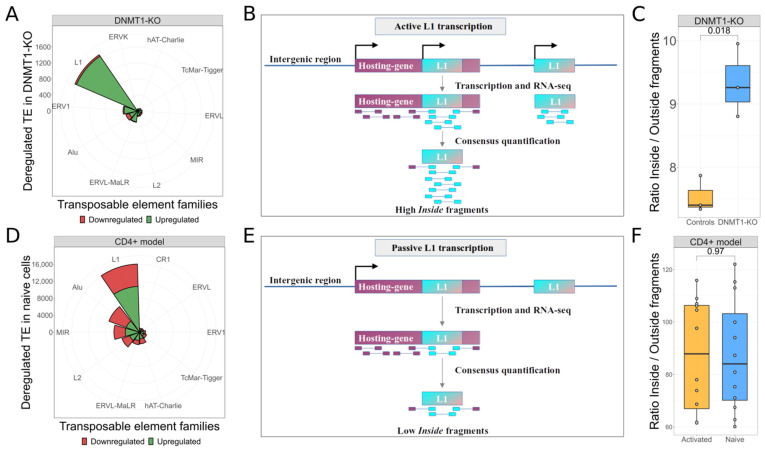
The KO of *DNMT1* gene leads to autonomous L1 transcription. (**A**). Top-10 deregulated TE families in *DNMT1* model. L1 is the most upregulated TE family with 1660 elements. (**B**). Rationale of our method for detecting autonomous transcription of L1 elements. Autonomous transcription of L1s will produce a higher amount of *Inside* fragments with both reads mapped inside the L1 consensus resulting in a higher *Inside/Outside* ratio. (**C**). Analysis to detect autonomous transcription of L1 elements. Upon the KO of *DNMT,* the upregulation of L1s derives by autonomous transcription of elements. (**D**). Top-10 deregulated TE families in naive T *CD4^+^* cells. L1 is the most upregulated TE family with 10,794 elements. (**E**). Rationale of our method for detecting non-autonomous transcription of L1 elements. Transcription of L1s embedded in other transcriptional units will produce a low amount of Inside fragments. (**F**). Analysis to detect autonomous transcription of L1 elements. In naive T CD4+ cells, the upregulation of L1s derives by a non-independent transcription of elements. Given that the two distributions are similar the overexpression of L1s is due to their transcription as part of other transcriptional units.

The first set of analyses was carried out to explore the transcriptomic changes of human neural progenitor cells (hNPCs) produced in the Jonsson’s study [30]. This dataset is composed of RNA-seq data derived from three controls and three samples in which the *DNMT1* gene was knocked-out with CRISPR-Cas9 technology. As Jonsson and colleagues demonstrated, the abrogated activity of the most important maintenance DNA methyltransferase [56] erases a fundamental epigenetic repressive layer from the L1 defense mechanisms. Aiming to confirm whether L1 elements were autonomously transcribed [57,58] in this model, we used *SQuIRE* [38] to quantify the locus-specific expression of TEs. Our differential expression analysis highlighted that the KO of *DNMT1* leads to a strong upregulation of TEs: 3015 TEs result upregulated and 277 downregulated. Interestingly, 1660 L1s were upregulated, resulting the most upregulated TE family in this experiment, as shown in Figure 1A.

Considering that ~99% of L1 RNA sequences in human arise from L1s embedded in other transcripts rather than from L1 promoters [59], we aimed to confirm the autonomous transcription of L1 elements in this model. In the method we developed (Figure 1B), paired-end reads are first aligned on the L1 consensus sequence and then on the human reference genome. The goal is to identify the number of fragments that are completely aligned inside the L1 elements (*Inside* fragments) and the number of fragments with a read aligned inside an L1 and the other one mapping outside the L1 on the reference genome (*Outside* fragments). We reasoned that the sequencing of L1 autonomous transcripts should produce mostly paired-end reads mapping within the internal part of L1 RNAs, hence mainly producing *Inside* fragments. Accordingly, by computing the *Inside/Outside* ratio, high ratio levels should result when L1 elements are autonomously transcribed. Applying our methodology to the *DNMT1* experiment, we were able to calculate a mean *Inside/Outside* ratio of 7.53 in the control group and a mean ratio of 9.33 in the KO group. A significantly (*t*-test *p*-value = 0.018) higher *Inside/Outside* ratio was observed in the *DNMT1* KO samples with respect to controls (Figure 1C), confirming that the observed upregulation of L1s is due to autonomous and independent L1 transcription and that the KO of *DNMT1* causes the transcriptional activation of genomic L1 loci.

In order to verify the capability of our method to identify situations in which L1 transcriptional upregulation is determined by non-autonomous and non-independent L1 transcription (i.e., L1s transcription mainly results from the transcription of their fragments as part of canonical genes), we took advantage of the Marasca et al. [31] dataset. In this study, the authors observed that naive CD4^+^ T cells transcribe L1-containing transcripts as non-canonical splicing variants. Upon cell activation, modifications in the splicing pattern induce the downregulation of these alternative transcripts promoting the canonical ones. We started quantifying the locus-specific expression of TE with *SQuIRE* [38]. Our differential expression analysis confirmed that naive cells, with respect to activated ones, are characterized by overexpression of L1 elements: 10,794 L1s are up-regulated and 5289 are down-regulated (Figure 1D). To assess if L1 overexpression is the result of L1s transcribed as part of other transcriptional units as Marasca et al. demonstrated, we applied our methodology to calculate the *Inside/Outside* ratio (Figure 1E). Applying our methodology to the *CD4^+^* experiment, we were able to calculate a mean *Inside/Outside* ratio of 87.43 in the naive group and a mean ratio of 87.12 in the active group. Since no statistically significant differences between the two groups were observed (Figure 1F), the *CD4^+^* model allowed us to confirm the lack of autonomous L1 transcription in naive T cells and therefore the reliability of our method to discriminate between the autonomous and non-autonomous transcription of L1 elements when upregulation of L1s is observed with standard procedures.

### 3.2. L1 Transcripts Could Act as ceRNA

**Figure 2:**
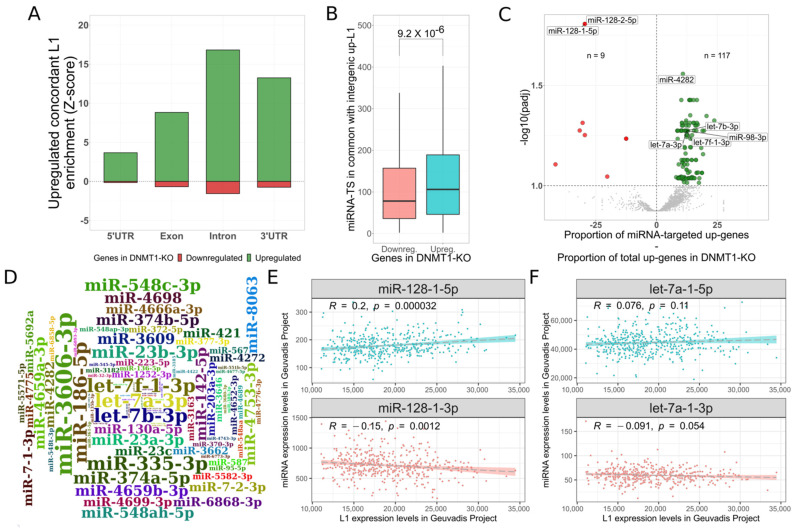
L1 transcripts might act as ceRNA. (**A**). Overlap analysis of L1s and protein-coding genes. Regions of upregulated genes are significantly enriched to contain upregulated L1 fragments. We found particularly interesting the strong enrichment in the 3′UTRs. (**B**). Analysis of miRNA target sites sharing between autonomously transcribed L1s and the 3′UTRs of protein-coding genes in *DNMT1* model. The upregulated genes share a significantly higher number of miRNA target sites with *active* L1s, adding support to a possible ceRNA activity of L1 transcripts. (**C**). Analysis to identify miRNAs sequestered by L1s. Each analyzed miRNA is represented by a point with the X-axis indicating the delta between the proportion of targeted upregulated genes and the total upregulated genes proportion. The Y-axis represents the -log10(FDR) of the proportional statistical test applied. The 117 miRNA in green represent the most probable pool of miRNAs that are undergoing the ceRNA activity from L1s. (**D**). In this wordcloud are shown the 117 miRNAs whose size font is proportional to the absolute number of experimentally validated target genes upregulated in *DNMT1* model. The let-7 family is among the top-10 miRNAs with the higher number of connections to these upregulated genes. (**E**). Correlation analysis between miR-128-1 (Y-axis) and L1 (X-axis) expression levels in Geuvadis dataset. Upon the L1 overexpression, the miR-128-1-5p levels concordantly increases probably as part of defense cellular mechanisms. (**F**). Correlation analysis between let-7a-1 (Y-axis) and L1 (X-axis) expression levels in Geuvadis dataset. No significant associations were found.

In order to inspect the transcriptional changes characterizing *DNMT1 KO* in the experiment by Jonsson and colleagues, we carried out differential gene expression analysis: 2188 genes resulted upregulated and 627 downregulated. Functional enrichment analyses revealed that genes belonging to the piRNA pathway (GO:0034587) [13] and to p53 transcriptional gene network (WP:WP4963) [60] were significantly enriched among the upregulated genes, while several proliferation-related genesets (e.g., GO:0042127) [61] were globally enriched among downregulated genes. These enrichments suggest that cells might be responding to transcriptional activation of L1s and possibly to a genotoxic stimulus [62]. Once the cell response was established, we performed an overlap analysis of genomic coordinates between L1 and the genic structures of protein-coding genes. The results revealed that upregulated genes are significantly enriched (Z-score > 3) to be in overlap with upregulated L1 elements in all the considered genic structures, as indicated in Figure 2A. The most intriguing finding was the observation that the 3′UTRs of upregulated genes were also significantly enriched to host upregulated L1 elements.

We then wondered about the existence of a possible ceRNA activity mediated by L1 transcripts. In our hypothesis, overexpressed L1 transcripts share miRNA target sites with the 3′UTRs of specific gene target sets. In this way, L1s might be able to recruit miRNAs from the target transcripts. As a result of this mechanism, transcripts from which miRNAs are sequestered should result upregulated. In order to verify this hypothesis, we selected the 799 upregulated extragenic L1s to represent the group of potentially autonomously transcribed L1 elements (referred to as *active* L1s). This set was chosen since they should less likely be involved in passive transcription as part of canonical transcripts [54], therefore reducing possible background noise from exonized L1s. We then identified the miRNA target sites both in the *active* L1s and in the 3′UTRs of protein-coding genes. In this pilot study we exclusively searched for exact matches to 8-mer seed-matched sites because it is believed to be the most effective single canonical site [19]. From this analysis, upregulated genes resulted to share on average 147 miRNA target sites with *active* L1s while the downregulated ones showed an average of about 114 miRNA target sites in common with the *active* L1s. The difference between the two groups is significant and indicates that upregulated genes, with respect to downregulated ones, have a significantly higher number of miRNA target sites in common with *active* L1s (Figure 2B, *p*-value = 2.35^−7^; Supplementary Table S1). This result suggests that the group of upregulated genes, sharing more miRNA target sites with autonomously transcribed L1s, might be subjected to a possible ceRNA activity mediated by upregulated L1s, adding support to our hypothesis.

In an attempt to identify the miRNAs that could bind *active* L1s, we hypothesized that genes usually targeted by these should result mostly upregulated. With this in mind, we analyzed 2563 miRNAs that were targeting at least one *active* L1s. For each miRNA, the protein-coding genes containing target sites were classified as up or down-regulated. Then, we searched for miRNAs in which the proportion of targeted upregulated genes was significantly (FDR < 0.1) higher than the proportion of downregulated ones. With this procedure, we identified 117 miRNAs that represent the most suitable pool of elements undergoing the putative ceRNA activity mediated by L1s (Figure 2C, Supplementary Table S2). Then, to understand which genes are putatively upregulated upon the L1 ceRNA activity, we used the MIENTURNET [51] tool. In this analysis, we provided the list of 117 identified miRNAs for discovering miRNA-genes interactions categorized in the miRTarBase database [52] as experimentally validated. The tool found 3878 miRNA-genes interactions involving 108 miRNAs and 543 genes. Of them, 57 were upregulated in the *DNMT1* model. The enrichment analysis of these 57 genes revealed that they are involved in processes that modulate transcription (GO:0006355) and particularly the p53 transcriptional program (WP4963). These results suggest that a putative L1-miRNA-transcript network might be involved in the regulation of genes that are part of a defense mechanism, specifically against L1 overexpression. Interestingly, four members of the let-7 family (let-7a-3p, let-7b-3p, let-7f-1-3p and miR-98-3p) [63], already known to target L1 transcripts, were found to be among the top-10 miRNAs with the highest number of connections to the upregulated genes (nine connections with respect to an average of 3.58 per miRNA, Figure 2D) which corroborates our hypothesis.

On the other hand, the strong enrichment for downregulated genes to share miR-128 targets with L1s seems to go against our hypothesis. MiR-128 has indeed been demonstrated to also target L1 transcripts [23], and therefore we were expecting to observe an enrichment in upregulated genes such as the one observed for members of the let-7 family. Instead, we observed the opposite with miR-128 resulting the most significant miRNA associated to downregulated genes (Figure 2C). In order to explain this difference, we took advantage of the Geuvadis dataset [34] which contains data from small RNAseq and RNAseq of lymphoblastoid cell lines from ~500 individual. These data allowed us to correlate the expression of all miRNAs (short RNAseq) with the expression of any other cellular transcript (RNAseq). We exploited them to evaluate the correlation between the expression of miR-128 and L1s and between let-7 and L1s. As shown in Figure 2E, the expression of the miR-128-1-5p results positively correlated (*p*-value = 0.000032) to L1 transcript levels. The putative passenger 3p strand results negatively correlated (*p*-value = 0.0012), reflecting its possible decay [64]. Conversely, as already observed [24], the expression levels of let-7-a-1 do not correlate with L1 transcript abundance (Figure 2F), suggesting that the expression of this miRNA can be interpreted as rather constant and independent by L1 expression levels. Overall, these results suggest that miR-128 levels increase when L1 transcript levels increase possibly as part of a response mechanism against TEs. The increased amount of miR-128 following L1 upregulation would then target not only L1 transcripts [65], but also, at least in part, canonical transcript targets of miR-128, causing their downregulation. This would not happen in the case of let-7, because its transcription does not change following L1 upregulation.

### 3.3. Support for L1 ceRNA Activity Using an Independent Experiment in Which a Specific L1 Is Artificially Overexpressed

**Figure 3:**
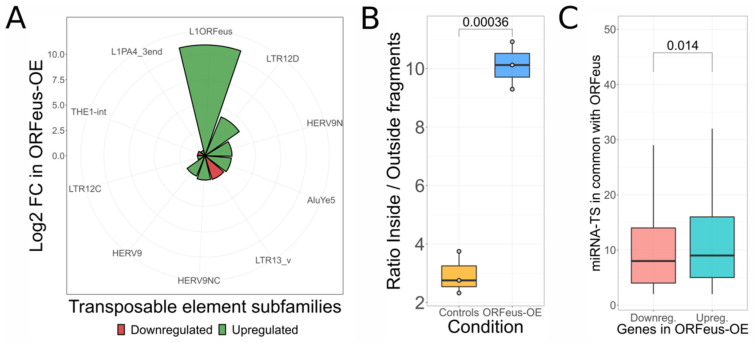
L1 could act as ceRNA when artificially overexpressed. (**A**). TE subfamilies significantly deregulated in the ORFeus-OE model. In this experiment, the L1 construct is the most upregulated TE with a log2 fold-change of 10.92. (**B**). Analysis to detect autonomous transcription of L1. In ORFeus-OE cells, the upregulation of L1 is deriving from an autonomous transcription of the ORFeus element. (**C**). Analysis of miRNA target sites sharing between artificial L1 construct and the 3′UTRs of protein-coding genes in ORFeus-OE model. The upregulated genes share a significantly high number of miRNA target sites with the L1 construct, reflecting a possible ceRNA activity of the artificial construct transcript.

To add support to our model, we analyzed the Ardeljan et al. [32] dataset composed of RNA-seq data derived from retinal pigment epithelial cells (RPE). In this cellular model, three samples overexpressing a codon-optimized L1 ORFeus construct were compared to three control samples. The aim of our analyses was to validate the activity of L1 as a ceRNA exploiting a cellular context perturbed exclusively by the overexpression of a single L1 without affecting DNA methylation. To assess the capability of *ORFeus-OE* model to overexpress the artificial L1 construct, we used the consensus-specific tool *TEspeX* [54], quantifying TE transcription levels. From the differential expression analysis, seven TE subfamilies were upregulated and two downregulated. The strongest (log2FC = 10.92) upregulated TE was the L1 construct overexpressed in the experiment, as visible in Figure 3A. To confirm the autonomous transcription of the L1 construct, our methodology was applied by using the L1 ORFeus sequence as consensus and without considering the genome mapping step in the pipeline (see Methods) because the expression construct is of exogenous origin. With this procedure, we calculated a mean *Inside/Outside* ratio of 2.94 in the control group and a mean of 10.11 in the ORFeus-OE samples. Hence, we observed a significantly (*t*-test, *p*-value = 0.00036) higher *Inside/Outside* ratio for the ORFeus-OE samples with respect to controls (Figure 3B) confirming the autonomous increase in the amount of L1 transcripts in this experimental setting.

To understand if the overexpressed L1 construct could be acting as ceRNA, we performed the differential gene expression analysis, identifying 3352 upregulated genes and 2890 downregulated ones. Then, we identified miRNA target sites in the L1 construct and in the 3′UTRs of protein-coding genes. Upregulated genes resulted to share an average of 10.38 miRNA target sites with the L1 construct. This is significantly higher with respect to the downregulated ones, showing an average of 9.38 (Figure 3C, *p*-value = 0.014). Moreover, in this case, our observations support the idea that the group of genes sharing a higher number of miRNA target sites with the L1 overexpressed construct could be undergoing ceRNA activity, resulting in their upregulation.

### 3.4. Putative L1 ceRNA Activity Might Depend on Autonomous L1 Transcription and AGO2

**Figure 4:**
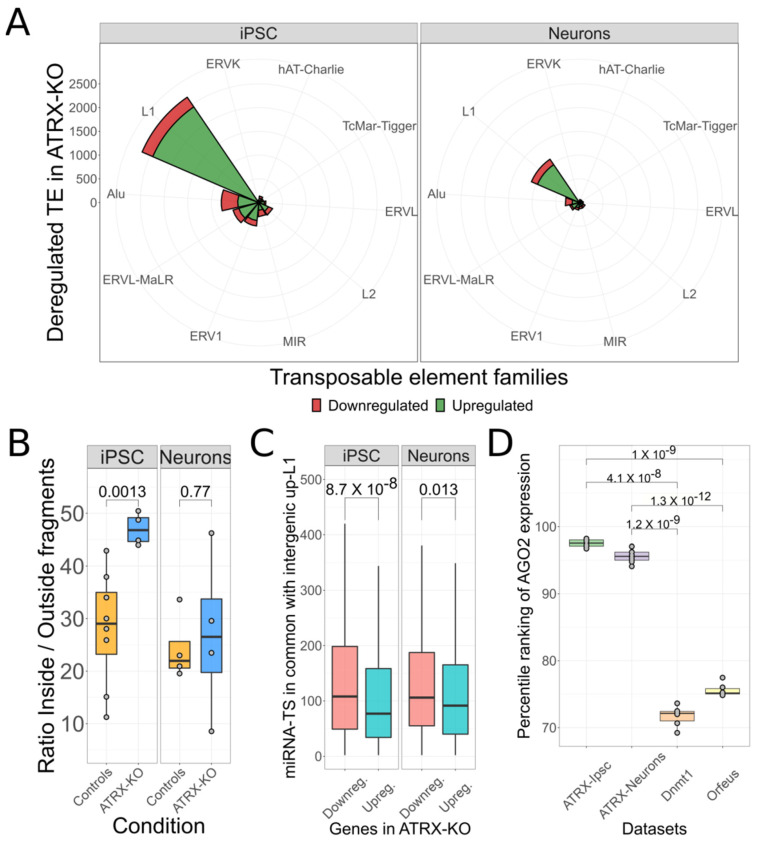
L1 ceRNA activity potentially relies on autonomous L1 transcription and Ago2 levels. (**A**). Top-10 deregulated TE families in *ATRX* model. Both in iPSC cells and in neurons, L1 family is the most upregulated TE family. (**B**). Analysis to detect autonomous transcription of L1 elements in *ATRX* model. Upon the KO of ATRX, the upregulation of L1 elements seems not to derive from an autonomous transcription of elements in neurons. (**C**). Analysis of miRNA target sites sharing between *active* L1s and the 3′UTRs of protein-coding genes in *ATRX* model. Downregulated genes show a higher number of miRNA target sites in common with overexpressed L1s. (**D**). Comparative analysis of Ago2 expression levels in all analyzed datasets.

Having collected evidence that overexpressed L1 transcripts might work as ceRNA, we decided to investigate other conditions leading to L1 deregulation. To this end, we analyzed a cellular model in which Deneault et al. [33] sequenced RNA of cells characterized by the KO of *ATRX* gene in reprogrammed human induced pluripotent stem cells (iPSC) and in iPSC differentiated in neuronal cells. *ATRX* is a chromatin remodeler gene that causes epigenetic modifications in retrotransposons *loci* [66]. To explore TE transcriptomic levels in the *ATRX* model, we used *SQuIRE* [38] to quantify the *locus*-specific expression of TEs. The differential expression analysis revealed a clear deregulation of TEs. Particularly, in iPSC cells the deregulation was more evident with 4490 up and 1217 down-regulated TEs, while the neuronal counterpart had 1820 up and 540 down-regulated elements. A common feature of both cell lines was that ~50% of upregulated TEs was represented by L1 elements: 2438 were the upregulated TEs in iPSC cells and 965 in neurons (Figure 4A). As observed in the *DNMT1* and *ORFeus-OE* models, in this experimental setting, the L1 is again the most upregulated TE family.

To investigate if the upregulation of L1s derived from a general active or passive TEs transcription, we applied our previously described method (Figure 4B). In iPSC cells, there was a clear difference in the *Inside/Outside* ratio between the control and the *ATRX*-KO groups (mean controls = 28.1 and mean *ATRX*-KO = 47.0). The neuronal cells, instead, were characterized by similar ratio between the two different groups (mean controls = 24.3 and mean *ATRX*-KO = 27.0). Therefore, while autonomous L1 transcription can be confirmed for iPSC cells (t-test, *p*-value = 0.0013), this is not the case for the neuronal cells (t-test, *p*-value = 0.77). These results suggest that neuronal cells carrying the *ATRX* KO, differently from the iPSC, might not undergo autonomous L1s upregulation. To explore the effects of L1 transcripts on the putative ceRNA dynamics, we identified the deregulated protein-coding genes. In iPSC, we found 818 up- and 1441 down-regulated genes while in neurons, they were respectively 319 and 369. We then compared miRNA target sites between the *active* L1s (1181 in iPSC and 479 in neurons) and the 3′UTRs of protein-coding genes. In contrast to *DNMT1* and *ORFeus-OE* models, the downregulated genes shared a significantly (*p*-value < 0.5) higher number of miRNA target sites with the *active* L1s in both experiments (Figure 4C). These results go against our hypothesis. While this could be explained for neuronal cells, since L1 transcripts could be passively transcribed as part of hosting genes which would decrease their capability to sequester miRNAs, we need a different explanation for the iPSC experiment.

The current understanding of the ceRNAs pathway indicates that the limiting factor for the ceRNA effect is the availability of Ago2 protein and not the amount of miRNA molecules. Indeed, it has been shown that a low amount of Ago2 is crucial for the ceRNA effect to take place while a high amount of it does not give raise to any target sites competition because there is no limitation of molecules for which to compete [67]. We therefore decided to investigate the expression levels of *AGO2* in a comparative manner between all the considered experiments. To make the expression levels of *AGO2* comparable among different experiments, we transformed the normalized counts related to *AGO2* expression in the corresponding percentile, as calculated with respect to the distribution of counts for all the transcripts relatively to each analyzed sample. From this analysis we observed that *AGO2* is expressed at a much higher level in the *ATRX* experiment (~95 percentile) with respect to the *DNMT1* and ORFeus (~73 percentile) ones (Figure 4D). These observations suggest that the reason why the *ATRX* experiment does not support a general L1s ceRNA effect could be due to the lack of a limiting dose of the Ago2 protein.

## 4. Discussion

L1 is the unique TE known to be still autonomously mobile in the human genome [1]. Although some elements are co-opted for cellular beneficial functions [6], deregulated L1 activity can be detrimental to cells [10]. As a result, cells use several mechanisms to regulate L1 transcript levels like the interfering activity of miRNAs [23]. Beyond this, TEs embedded in lncRNA are supposed to provide miRNA target sites competing with target genes for the same pool of miRNAs [29]. Despite the increasing interest in this field, a full comprehension of the regulatory layers in which L1 transcripts are involved is still lacking. Furthermore, it remains unclear how their expression levels are regulated and how their deregulation might impact transcriptional programs. In this work, we reanalyzed RNA-seq data of cells carrying mutations in genes affecting L1 transcript levels or overexpressing a specific L1 construct.

Analyzing the *DNMT1-*KO model [30], we confirmed a strong transcriptional activation of L1 loci following global demethylation. The upregulated protein-coding genes were enriched to contain L1 element fragments especially in their introns and 3′UTRs. This made us speculate that L1 transcripts might have ceRNA activity, competing with miRNAs normally targeting genes, resulting in upregulation. To support this hypothesis, we demonstrated that the upregulated genes, with respect to the downregulated ones, share a higher number of miRNA target sites with the autonomously transcribed L1 elements. This feature is in support of a possible L1 ceRNA activity. In our model, overexpressed L1 transcripts sharing more miRNA target sites with the 3′UTR of a given set of transcripts are prone to recruit miRNAs from them. As a consequence, transcripts from which more miRNAs are sequestered result upregulated. Identifying the putatively sequestered miRNAs, we found a pool of 117 miRNAs including different miRNAs belonging to the let-7 family. This family is experimentally validated to bind the ORF2 of L1 transcripts [24], supporting the reliability of our experimental procedure for identifying miRNAs sequestered by L1s. Analyzing the genes that should be upregulated upon L1 ceRNA activity, we found an enrichment for genes involved in the p53 transcriptional gene network. p53 is a tumor suppressor gene that induces transcriptional programs for responding to a variety of stress signals. Among these, direct [60] or indirect [68] functions are shown to control L1 activity. The L1 ceRNA activity might be a mechanism that induces a cellular response, like p53 targets transcription, which in turn act against L1 when these elements are overexpressed. The competition between L1 transcripts and canonical transcripts for the binding of a selected pool of miRNAs, might cause the transcriptional activation of genes that coherently work to repress L1 activities, protecting the well-being of cells. In this experiment, we also found an enrichment for downregulated genes to share miR-128 targets with L1s, which goes against our hypothesis since also this miRNA has been demonstrated to target L1s. Nevertheless, our model still holds if we take into account that the expression of miR-128 might increase or be induced in *DNMT1-*KO cells, which is reasonable because the overexpression of miR-128 can also, at least in part, justify the increase we have observed in the expression of p53 target-genes [69]. In addition, the analysis of Geuvadis dataset clearly shows a positive correlation between miR-128 expression and L1 transcript levels in lymphoblastoid cell lines, suggesting that, conversely to let-7 miRNAs whose transcription does not change, an induction in the expression of mir-128 happens in response to L1 overexpression.

We then analyzed an artificial condition with L1 RNA overexpression driven from a synthetic plasmid [32]. Further, in this case, we confirmed that upregulated genes are enriched for genes sharing a higher number of miRNA targets with the overexpressed L1 adding further support to a possible L1 ceRNA activity.

As a third study with overexpression of L1s, we analyzed a dataset derived from knocking out the *ATRX* gene in cultured neurons and iPSC cell lines as a model of autism [33]. Initially, an enrichment was found for shared miRNA targets among genes downregulated upon *ATRX-*KO. While these data were in contrast with our hypothesis, we took then into account that the ceRNA effect requires that Ago2 protein is expressed in limiting amounts. When Ago2 is in high quantity, the competing effect does not manifest because other molecules such as miRNAs are generally present in non-limiting abundance [67,70]. Reassuringly, the amount of *AGO2* mRNA in the *ATRX-*KO cell model was significantly higher with respect to those in the *DNMT1-*KO and in the Orfeus construct experiments, thus further supporting our model.

In conclusion, we have shown that cellular conditions, with strong autonomous L1 transcription, are characterized by the sharing of miRNA target sites between overexpressed L1s and upregulated genes when Ago2 availability is limited. This sharing might be at the basis of a competition for miRNAs targeting. The sequestering of miRNAs by L1 transcripts could then result in the upregulation of a given set of transcripts. Thus, our study provides initial evidence in support of the hypothesis of transposon acting as ceRNAs. In this model, the ceRNA activity might even result as a way for the cell to trigger defense mechanisms, such as the p53 transcriptional program, when the L1 transcript levels overcome a certain threshold.

Our results and evidence have been so far produced exclusively in-silico, and therefore specific validation of the model herein proposed is needed to ascertain the pervasive activity of TEs at the ceRNAs level. Once experimentally validated, we believe that our hypothesis will help future studies for dissecting cellular responses in both developmental [71] and pathological [10] conditions characterized by the transcriptional activation of L1 elements.

## Data Availability

RNA-seq data from *DNMT1*-KO used in this study are available in the ENA-EBI repository (Accession code: PRJNA420729) https://www.ebi.ac.uk/ena/browser/view/PRJNA420729 (accessed on 19 November 2020); RNA-seq data from CD4^+^ used in this study are available in the ENA-EBI repository (Accession code: PRJEB41930) https://www.ebi.ac.uk/ena/browser/view/PRJEB41930 (accessed on 8 March 2022); RNA-seq data from Geuvadis project used in this study are available in the ENA-EBI repository (Accession code: PRJEB3366) https://www.ebi.ac.uk/ena/browser/view/PRJEB3366 (accessed on 19 November 2020); Short RNA-seq data from Geuvadis project used in this study are available in the ArrayExpress repository https://www.ebi.ac.uk/arrayexpress/files/E-GEUV-3/analysis_results/GD452.MirnaQuantCount.1.2N.50FN.samplename.resk10.txt (accessed on 27 October 2022); RNA-seq data from ORFeus used in this study are available in the ENA-EBI repository (Accession code: PRJNA491205) https://www.ebi.ac.uk/ena/browser/view/PRJNA491205 (accessed on 19 July 2021); RNA-seq data from *ATRX*-KO used in this study are available in the ENA-EBI repository (Accession code: PRJNA422099) https://www.ebi.ac.uk/ena/browser/view/PRJNA422099 (accessed on 18 September 2021).

## Supplementary Materials

The following supporting information can be downloaded at: https://www.mdpi.com/article/10.3390/biomedicines10123279/s1, Table S1: Information about miRNA target sites in common between protein-coding genes and intergenic upregulated L1 in *DNMT1* experiment; Table S2: Number of deregulated target genes for each analyzed miRNA in *DNMT1* experiment.
